# *In Vitro* Biologic Activities of the Antimicrobials Triclocarban, Its Analogs, and Triclosan in Bioassay Screens: Receptor-Based Bioassay Screens

**DOI:** 10.1289/ehp.11200

**Published:** 2008-05-16

**Authors:** Ki Chang Ahn, Bin Zhao, Jiangang Chen, Gennady Cherednichenko, Enio Sanmarti, Michael S. Denison, Bill Lasley, Isaac N. Pessah, Dietmar Kültz, Daniel P.Y. Chang, Shirley J. Gee, Bruce D. Hammock

**Affiliations:** 1 Department of Entomology and Cancer Research Center; 2 Department of Environmental Toxicology; 3 Center for Health and Environment; 4 Department of Molecular Biosciences and Center for Children’s Environmental Health and Disease Prevention; 5 Department of Animal Science and; 6 Department of Civil and Environmental Engineering, University of California, Davis, Davis, California, USA

**Keywords:** androgen receptor, antimicrobial, aryl hydrocarbon receptor, bioactivity, carbanilide analog, estrogen receptor, ryanodine receptor, sensitization, signal amplification, triclocarban, triclosan

## Abstract

**Background:**

Concerns have been raised about the biological and toxicologic effects of the antimicrobials triclocarban (TCC) and triclosan (TCS) in personal care products. Few studies have evaluated their biological activities in mammalian cells to assess their potential for adverse effects.

**Objectives:**

In this study, we assessed the activity of TCC, its analogs, and TCS in *in vitro* nuclear-receptor–responsive and calcium signaling bioassays.

**Materials and methods:**

We determined the biological activities of the compounds in *in vitro*, cell-based, and nuclear-receptor–responsive bioassays for receptors for aryl hydrocarbon (AhR), estrogen (ER), androgen (AR), and ryanodine (RyR1).

**Results:**

Some carbanilide compounds, including TCC (1–10 μM), enhanced estradiol (E_2_)-dependent or testosterone-dependent activation of ER- and AR-responsive gene expression up to 2.5-fold but exhibited little or no agonistic activity alone. Some carbanilides and TCS exhibited weak agonistic and/or antagonistic activity in the AhR-responsive bioassay. TCS exhibited antagonistic activity in both ER- and AR-responsive bioassays. TCS (0.1–10 μM) significantly enhanced the binding of [^3^H]ryanodine to RyR1 and caused elevation of resting cytosolic [Ca^2+^] in primary skeletal myotubes, but carbanilides had no effect.

**Conclusions:**

Carbanilides, including TCC, enhanced hormone-dependent induction of ER- and AR-dependent gene expression but had little agonist activity, suggesting a new mechanism of action of endocrine-disrupting compounds. TCS, structurally similar to noncoplanar *ortho*-substituted poly-chlorinated biphenyls, exhibited weak AhR activity but interacted with RyR1 and stimulated Ca^2+^ mobilization. These observations have potential implications for human and animal health. Further investigations are needed into the biological and toxicologic effects of TCC, its analogs, and TCS.

The antimicrobial agents triclosan (TCS; 2,4,4′-trichloro-2′-hydroxydiphenyl ether) and triclocarban (TCC; 3,4,4′-trichloro-carbanilide) are high-production-volume chemicals that are widely used as “value added” chemicals in personal care products. In a limited retail survey, approximately 45% of liquid and bar soaps on the market contained these antimicrobials; TCS and TCC were the predominant antimicrobials in liquid soaps and bar soaps, respectively (Perencevich et al. 2001). According to the U.S. Environmental Protection Agency (2007), U.S. consumers spend nearly $1 billion/year on these products. TCS is a broad-spectrum bacteriostatic germicide that is now used in consumer products such as liquid hand soap, toothpaste, mouth rinse, cosmetics, pharmaceutical products, fabrics, plastics, textiles, and plastic kitchenware since its original introduction as an active ingredient in a surgical scrub for professional health care in 1972 (Tierno 1999). It is a powerful antibacterial agent that inhibits the activity of the enzyme enoylacyl carrier-protein reductase, which catalyzes an essential step in membranes of many bacteria and fungi (Heath et al. 1999; McMurry et al. 1998b). TCC, another antimicrobial agent, is more often added to consumer bar soaps and deodorants, and it is active predominantly against gram-positive bacteria (McDonnell and Russell 1999). The carbanilide analog 3-trifluoromethyl-4,4′-dichlorocarbanilide (TFC) is also used as an antibacterial agent (Jeffcoat et al. 1977).

Although these compounds are broadly classified as halogenated aromatic hydrocarbons, TCS has functional moieties representative of phenols, diphenyl ethers, and polychlorinated biphenyls (PCBs), whereas TCC is structurally related to carbanilide compounds, including some drugs and pesticides, and sterically and electronically related to a variety of other chemicals (Figure 1). Both TCS and TCC have been detected at microgram per liter levels in waterways in the United States and Switzerland, indicating extensive contamination of aquatic ecosystems (Halden and Paull 2005; Kolpin et al. 2002; Lindstrom et al. 2002). The potential of these compounds for bioaccumulation has raised public concern regarding their possible effects on human health (Coogan et al. 2007; Darbre 2006; Daughton and Ternes 1999) and microbial resistance (McMurry et al. 1998a). Recent reports note that TCS levels as high as 2,000 μg/kg lipid have been detected in human breast milk (Dayan 2007), and concentrations in human fluids such as plasma and milk are positively correlated to levels of exposure (Adolfsson-Erici et al. 2002; Allmyr et al. 2006; Dayan 2007; Hovander et al. 2002).

Because of these concerns, we screened TCS, TCC, and a series of TCC analogs for biological activity in several mechanistically derived cell-based assay systems. Mammalian ligand-dependent nuclear receptors serve as biomarkers that evaluate the potential of an environmental toxicant to affect endocrine and non–endocrine-signaling systems. One set of assays used in the present study is based on the chemically activated luciferase gene expression (CALUX) bioassays. The recombinant cells used in the CALUX bioassays include a stably transfected aryl hydrocarbon receptor (AhR)-, androgen receptor (AR)-, or estrogen receptor (ER)-responsive firefly luciferase reporter gene that responds to chemicals that can bind to and/or activate the respective receptor, leading to the induction of luciferase reporter gene expression.

AhR is a transcription factor that activates gene expression in a ligand-dependent manner. Exposure to the most potent ligand, 2,3,7,8-tetrachlorodibenzo-*p*-dioxin (TCDD), results in a variety of toxic and biological responses, most of which are AhR dependent, such as birth defects, immunotoxicity, tumor production, changes in metabolism, and lethality. Dioxin-like PCBs, polychlorinated dibenzofurans, and related chemicals that mimic the action of TCDD at the level of the AhR are detected by measurement of their ability to stimulate AhR-dependent gene expression in the CALUX bioassay (Garrison et al. 1996; Han et al. 2004).

Steroid hormones control reproduction, metabolism, and ion balance in vertebrates. ER and AR are nuclear receptors for estrogenic and androgenic chemicals such as estradiol (E_2_) and testosterone, respectively, that function as transcription factors to regulate female and male reproduction, sexual development, and bone structure. Chemicals acting as endocrine-disrupting compounds (EDCs) affect these receptors and lead to activation/ inhibition of hormone-dependent gene expression. However, EDCs may also alter hormone receptor function simply by changing phosphorylation of the receptors (activating them) without the responsible chemical or natural ligand ever binding to the receptor (Weigel and Moore 2007). Recently, Chen et al. (2008) reported data indicating a mechanism of endocrine disruption that involves the receptor but that does not appear to act through competition with the receptor’s primary binding site. Instead, they observed an amplification or enhancement of the ability of the chemical to stimulate gene expression in concert with the natural ligand, possibly indicating a new type of EDC that may not share the basic qualities of previously defined EDCs.

An increasing number of studies report that chemicals in the environment, mimicking natural estrogen, interact with or affect the ER in cells and thereby disrupt normal endocrine function, raising public concerns about the biological/reproductive effects of these chemicals (Darbre 2006). Reproductive health concerns regarding androgenic EDCs that may reduce sperm production, alter genital development, and contribute to neurologic syndromes in males have been proposed (Chen et al. 2007; Larsson et al. 2006; Sonneveld et al. 2005). In relation to the work reported here, TCS may have cytotoxic effects on breast cancer cells (Liu et al. 2002) and have endocrine-disrupting properties (antiandrogenic activity and thyroid-hormone-like activity) in aquatic species and human recombinant cells in culture (Chen et al. 2007; Foran et al. 2000; Veldhoen et al. 2006).

The ryanodine (Ry) receptor type 1 (RyR1)-based bioassay is used for screening potential compounds exhibiting biological activity that alters Ca^2+^ homeostasis (Pessah et al. 2006). RyRs function as high-conductance Ca^2+^ channels broadly expressed in animal cells, including muscle (skeletal, cardiac, smooth), neurons, and immune cells. Chemicals that enhance or inhibit RyR channel activity, such as caffeine and Ry, have been demonstrated to influence Ca^2+^ signaling events and Ca^2+^-dependent processes in a number of cell types. Noncoplanar, *ortho*-substituted PCB congeners that exhibit weak AhR activity enhance the sensitivity of RyRs to activation by endogenous ligands in a manner requiring the immunophilin FKBP12–RyR complex (Wong et al. 1997). Disruption of Ca^2+^ homeostasis in the affected regions of the brain by compounds altering RyR may contribute to alteration of neurodevelopment and neuroplasticity function (Gafni et al. 2004; Wong et al. 2001).

The screening assays used in this study are part of a library of techniques developed by the University of California, Davis Superfund Basic Research Program, whose aim is to identify biomarkers of exposure and effect of toxic substances. The goal of this study was to demonstrate that such mechanistic, nuclear-receptor–based screening assays can rapidly provide useful information on environmental chemicals, and to assess the potential for the antimicrobials TCC, its analogs, and TCS to produce specific toxic effects that would warrant further study.

## Materials and Methods

### Chemicals

**Figure 1:**
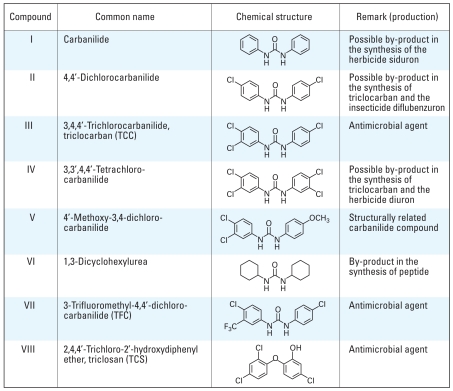
Chemical structures and use of TCC, its analogs, and TCS.

Compounds, identified by Roman numerals in the text, are listed in Figure 1; compounds were either purchased or synthesized in this laboratory. The carbanilide compounds, carbanilide (I; reported purity, 99.9%), TCC (III; reported purity, 99.3%), and 1,3-dicyclohexylurea (VI; reported purity, >98%) were purchased from Aldrich (St. Louis, MO); 3-trifluoromethyl-4,4′-dichloro-carbanilide (VII; reported purity, > 99%) was purchased from Chembridge (San Diego, CA). We synthesized structurally related carbanilide compounds (II, IV, and V; purity, > 99%) by condensing the appropriate isocyanate and amine according to previously published reports (Morisseau et al. 1999; Newman et al. 2001). We purchased TCS (VIII; reported purity of 99.8%) from Fluka (St. Louis, MO). The commercial TCC (III) and TCS (VIII) compounds were further purified to approximately 100% purity by recrystallization three times from ethanol and petroleum ether, respectively. We obtained TCDD from S. Safe (Texas A&M University, College Station, TX). We purchased dimethyl sulfoxide (DMSO), 17β-E_2_, and phenol red-free Dulbecco’s modified Eagle medium (DMEM) from Sigma Chemical Co. (St. Louis, MO); cell culture reagents and media from Gibco/BRL (Grand Island, NY); and 17β-testosterone from Alltech (State College, PA). All test compounds, except for the steroids, were dissolved in DMSO; steroids were dissolved in absolute ethanol. We purchased [^3^H]Ry (60–90 Ci/mmol; > 99% pure) from Perkin-Elmer New England Nuclear (Wilmington, DE) and unlabeled Ry (> 99% by ultraviolet-HPLC from Calbiochem (San Diego, CA).

### Cell-based AhR-mediated bioassay

Recombinant rat hepatoma (H4L1.1c4) cells were grown and maintained as previously described (Garrison et al. 1996). These cells contain the stably integrated, dioxin-responsive–element (DRE)-driven firefly luciferase reporter gene plasmid pGudLuc1.1. Transcriptional activation of the plasmid occurs in a ligand-, dose-, and AhR-dependent manner. Cells were plated into white, clear-bottomed 96-well tissue culture dishes at 75,000 cells/well and allowed to attach for 24 hr. Cells were incubated with carrier solvent DMSO (1% final solvent concentration), TCDD (1 nM), the indicated compound (for measurement of agonist activity), or the indicated compound plus 1 nM TCDD (for measurement of antagonist activity) for 4 hr at 37°C. For luciferase measurement, sample wells were washed twice with phosphate-buffered saline, followed by addition of cell lysis buffer (Promega, Madison, WI); the plates were then shaken for 20 min at room temperature to allow cell lysis. We measured luciferase activity in each well using a Lucy2 microplate luminometer (Anthos, Durham, NC) with automatic injection of Promega stabilized luciferase reagent. Luciferase activity in each well was expressed relative to that maximally induced by TCDD.

### Cell-based ER-mediated bioassay

Recombinant human ovarian cancer cells (BG1Luc4E_2_, ER-α–positive) were grown and maintained as previously described (Rogers and Denison 2000). These cells contain a stably integrated, ER-responsive firefly luciferase reporter plasmid, pGudLuc7ERE. Cells were maintained in estrogen-stripped media for 5 days before they were plated into white, clear-bottomed 96-well tissue culture dishes at 75,000 cells/well and allowed to attach for 24 hr. Cells were then incubated with carrier solvent (ethanol; 1% final solvent concentration), E_2_ (1 nM), the indicated compound (for measurement of agonist activity), or the indicated compound plus 1 nM E_2_ (for measurement of antagonist activity) for 24 hr at 37°C. We measured luciferase activity as described above; activity is expressed relative to that maximally induced by E_2_.

### Cell-based AR-mediated bioassays

For the cell-based human AR-responsive bioassay, recombinant human cells [T47D-androgen-responsive element (ARE)] were grown and maintained as described above for H4L1.1c4 cells. The T47D-ARE cells contain a stably integrated AR-responsive firefly luciferase reporter gene plasmid, pGudLuc7ARE (Rogers and Denison 2002). Cells were plated into white, clear-bottomed 96-well tissue culture dishes at 75,000 cells/well and allowed to attach for 24 hr. Cells were incubated with carrier solvent (ethanol; 1% final solvent concentration), 10 μM testosterone, the indicated compound (for measurement of agonist activity), or the indicated compound plus the indicated concentration of testosterone (for measurement of antagonist activity) for 24 hr at 37°C. We measured luciferase activity as described above; activity in each well is expressed relative to that maximally induced by testosterone. For comparison, we present data from a previously published study on the effect of these chemicals on AR in human embryonic kidney (HEK) 2933Y cells that lack key steroid-metabolizing enzymes (Chen et al. 2008).

### RyR1-mediated bioassay

Preparation of primary cultures of skeletal myotubes from wild-type mice has been previously described (Rando and Blau 1994). Wild-type myoblasts were cultured in treated dishes coated with calf skin collagen with F-10 nutrient medium containing 20% (vol/vol) fetal bovine serum, 2 mM l-glutamine, 4 ng/mL fibroblast growth factor, 100 units/mL penicillin-G, and 100 μg/mL streptomycin sulfate at 37°C in 10% CO_2_/5% O_2_. For fura-2 imaging, cells were plated onto 96-well μ-clear plates (Greiner Bio-One GmbH, Monroe, NC) coated with Matrigel (Becton Dickinson, Franklin Lakes, NJ). Upon reaching ~ 80% confluency, the cells were differentiated into multinucleated myotubes over a period of 3 days using DMEM containing 5% (vol/vol) heat-inactivated horse serum, 2 mM l-glutamine, penicillin-G, and streptomycin sulfate at 37°C in 10% CO_2_/10% O_2_. Intracellular Ca^2+^ signals were recorded as previously described (Fessenden et al. 2000). Briefly, differentiated primary wild-type myotubes were loaded with the calcium indicator dye fura-2, and the cells were imaged using an intensified charge-coupled device camera.

The [^3^H]Ry-binding assay was performed as previously described (Pessah et al. 1987). Observation of [^3^H]Ry binding to the isolated sarcoplasmic reticulum preparation was performed in the presence of 1 nM [^3^H]Ry and 10 μM Ca^2+^. The binding reaction was carried out at 37°C for 3 hr in 0.5 mL containing 50 μg sarcoplasmic reticulum protein. We assessed nonspecific binding in the presence of 10 μM unlabeled Ry. We separated bound and free ligand by rapid filtration through Whatman GF/B glass fiber filters, using a Brandel (Gaithersburg, MD) cell harvester. Filters were washed with 2 volumes of 5 mL ice-cold wash buffer containing 20 mM Tris-HCl, 250 mM KCl, 15 mM NaCl, and 50μM CaCl_2_ (pH 7.1) and placed in vials with 5 mL scintillation cocktail (Ready Safe, Beckman Instruments, Fullerton, CA). We quantified [^3^H]Ry remaining on the filters by liquid scintillation spectrometry.

### Data analysis

We measured luciferase activity per well as relative light units. We calculated luciferase induction as a percentage of TCDD (AhR bioassay), E_2_ (ER bioassay), or testosterone (AR bioassay) activity by setting the maximal induction by 1 nM TCDD, 1 nM E_2_, or 10 μM testosterone at 100%. Background activity present in the vehicle control was subtracted from treated cells. For antagonistic effects in the AhR bioassay, the induction at 1 nM concentration of TCDD was set at 100%. When evaluating for enhancement effects of the hormone receptors in the CALUX bioassays, the induction at a concentration of 1 nM E_2_ (ER bioassay) or 10 μM testosterone (AR bioassay) was set at 100%, and the degree of enhancement by each compound tested was calculated as the ratio of the luciferase reporter gene induction value of each compound when combined with E_2_ or testosterone relative to that of the hormone alone. We calculated the fold increase of [^3^H]Ry binding by TCS in skeletal muscle sarcoplasmic reticulum vesicles by dividing the mean value of [^3^H]Ry binding triggered by TCS with that triggered by the solvent control (DMSO); the solvent had none of the effects seen with TCS. Values shown are mean ± SD from three independent experiments for each dose tested, with vehicle control values subtracted. We analyzed data by one-way analysis of variance, followed by a multiple comparisons test when appropriate, using SigmaStat version 3.5 (Systat Software, San Jose, CA). We set the level of significance at *p* < 0.05.

### Molecular modeling of TCS

We performed molecular modeling using the CS ChemOffice 2005 software package (CambridgeSoft Corp., Cambridge, MA). We compared the optimized geometries of 2,2′,3,5′,6-pentachlorobiphenyl (PCB-95) and TCS at their minimum energy levels with a minimum root mean square gradient of 0.1 computed by MM2 force fields. We measured the dihedral angles formed by two phenyl rings in the structures of PCB-95 and TCS after molecular modeling; three-dimensional projections of the structures of TCS and PCB-95 were simulated using ChemIDplus (National Library of Medicine 2008).

## Results

### Cell-based AhR-mediated bioassay

We evaluated the activity of AhR-mediated cells by measuring luciferase activity induced by test compounds compared with that of the solvent control (DMSO) or TCDD as positive control. As shown in Table 1, no carbanilide compounds tested (I–VII) exhibited induction except 1,3-dicyclohexylurea (VI), which induced reporter gene activity to 51% of that induced by TCDD. Interestingly, induction by compound VI was lower at the higher concentration, suggesting that it may be toxic to the cells, although we observed no overt cell toxicity by visual inspection. Except for compound VI, all carbanilides at higher concentrations (10 μM) inhibited TCDD-dependent luciferase gene expression between 20% and 70%, suggesting that these chemicals may act as weak AhR antagonists.

We tested TCS (compound VIII) in the AhR bioassay because of its structural similarity to hydroxylated metabolites of the polybrominated diphenyl ethers 2,4,4′-tribromodiphenyl ether [bromodiphenyl ether-28 (BDE-28)], and 2,2′,4,4′-tetrabromodiphenyl ether (BDE-47). TCS, at 10 μM, not only induced luciferase expression to 40% of that of TCDD induction but also inhibited the induction of luciferase expression by TCDD by approximately 30%. These agonist/antagonist results are consistent with TCS being a partial agonist of the AhR.

### Cell-based ER- or AR-mediated bioassay

**Figure 2:**
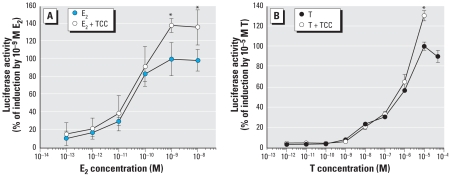
Results of ER- and AR-mediated bioassays showing the effects of 1 μM TCC on gene expression of ER (*A*) and AR (*B*) induced by E_2_ in BG1-ERE cells and testosterone (T) in T47D-ARE cells, respectively. Luciferase activity (mean ± SD) is expressed relative to that maximally induced by E_2_ and T in (*A*) and (*B*), respectively. *Significantly greater than E_2_ or T positive control groups (*p* < 0.05).

We evaluated activity of the recombinant ER-or AR-responsive cells by measuring luciferase activity induced by E_2_ or testosterone, respectively, and compared results from the carbanilide compounds with solvent controls or positive controls (E_2_, testosterone). Coincubation of E_2_ and TCC resulted in enhanced E_2_-dependent induction of luciferase gene expression, with significant increases observed at 1–10 nM E_2_ (Figure 2A). We also examined the effect of TCC on the ability of testosterone to induce AR-mediated reporter gene activity; similar to results with the ER-reporter system, TCC enhanced testosterone-dependent induction of luciferase gene expression in T47D-ARE cells, but only at the highest concentration (10 μM) of testosterone (Figure 2B). Amplification of testosterone-dependent induction of ARE-linked luciferase reporter gene in a stably transfected HEK 293-ARE cell line has been previously published (Chen et al. 2008), although that study reported the enhancement effect to occur at testosterone concentrations as low as 0.1 nM. Together, these results demonstrate that TCC can exert an enhancing effect on at least two members of the steroid hormone receptor family of transcription factors. Whether other related receptors will be similarly affected remains to be determined.

**Figure 3:**
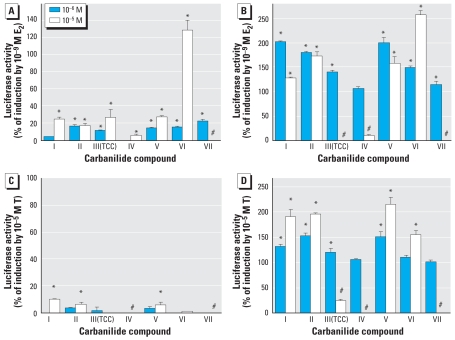
Effects of carbanilide compounds at 1 (10^−6^ M) and 10 μM (10^−5^ M) on ER- and AR-mediated activity in the absence (*A* and *C*) and presence (*B* and *D*) of E_2_ (1 nM; *A* and *B*) or testosterone (T; 10 μM; *C* and *D*) in ER-responsive (BG1-ERE cells) or AR-responsive (T47D-ARE cells) bioassay. *Significantly greater than the solvent control (*A* and *C*) or E_2_ or T positive controls (*B* and *D*) at *p* < 0.05 for agonist/amplification evaluation. ^#^Cell toxicity at 10-μM concentration.

The activity of TCC in the ER- and AR-responsive cells provides an interesting mechanism to enhance the endocrine-disrupting activity of chemicals. To determine whether other carbanilides also exert similar hormone-enhancing activity and whether they have any estrogenic or androgenic activity, we examined the ability of these chemicals to induce ER- or AR-dependent luciferase reporter gene activity and to enhance/inhibit hormone (E_2_/T)-dependent reporter gene induction in the cell bioassays. As shown in Figure 3A, TCC and its analogs, at concentrations of 1 or 10 μM, exhibited weak ER activity, < 30% of maximal E_2_-induced reporter gene induction; dicyclohexylurea (VI) induced ER-dependent gene expression only at 10 μM. Interestingly, compound VI at 10 μM induced ER-dependent reporter gene expression to a level significantly greater than that of a maximally inducing concentration of E_2_. The results of the combined treatment of the carbanilides and E_2_ (Figure 3B) revealed an enhancement of E_2_-dependent gene expression by several compounds, with some being more effective enhancers at the lower concentration (I, III, and V) and one (VI) being a more effective E_2_ enhancer at the higher concentration, increasing maximal E_2_-dependent induction by 2.5-fold. The dramatic reduction in E_2_-dependent induction of luciferase by compounds III, IV, and VII at 10 μM resulted from cell toxicity, as determined by visual inspection.

**Figure 4:**
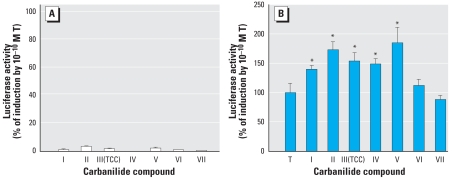
Effects of 1-μM carbanilide compounds on the AR-mediated activity in the absence (*A*) and the presence (*B*) of testosterone (T; 0.1 nM) in the HEK-2933Y AR-responsive cell system. The activity by carbanilide compounds I–V was reported by Chen et al. (2008). *Significantly greater than the solvent control (*A*) or T positive control (*B*) at *p* < 0.05 for agonist/amplification evaluation.

Examination of the ability of the carbanilide compounds to induce AR-dependent reporter gene activity (Figure 3C) revealed that most compounds either were inactive or had very low agonist activity (inducing luciferase activity to < 10% of a maximal inducing concentration of testosterone). When combined with testosterone (10 μM), the four carbanilide compounds (I, II, III, and V) at 1 μM enhanced testosterone-dependent gene expression, whereas compounds I, II, V, and VI at 10 μM enhanced luciferase gene induction to a significantly greater degree than observed with 1 μM (Figure 3D). The reduction in testosterone-dependent induction of luciferase activity by compounds III, IV, and VII at 10 μM resulted from cell toxicity as determined by microscopic examination. At 1 μM, compounds IV and VII did not enhance testosterone-dependent reporter gene expression. As shown in Figure 4A, all test compounds were inactive in HEK 2933Y-ARE cells; however, when combined with testosterone at a physiologically relevant concentration of 0.1 nM in HEK 2933Y-ARE cells (Figure 4B), the 1-μM concentration of the carbanilide compounds, except for TFC (VII), exhibited a range of 20–100% amplification of testosterone-induced AR activity. Carbanilide VI and VII did not alter the signal significantly. Taken together, the results observed in the two cell lines strongly support the ability of a number of the carbanilide compounds to interact with the AR or AR-signal transduction pathway, leading to enhancement of AR-dependent gene expression.

**Figure 5:**
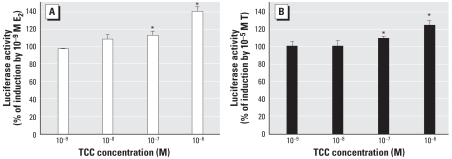
The effects of TCC (III) tested at different concentrations on ER (*A*) or AR (*B*) gene expression in the presence of steroid hormone E_2_ (1 nM) or testosterone (T; 10 μM) at a constant concentration in the ER-responsive (BG1-ERE; *A*) or AR-responsive (T47D-ARE; *B*) bioassay. *Significantly greater than the E_2_ or T positive control at *p* < 0.05.

**Figure 6:**
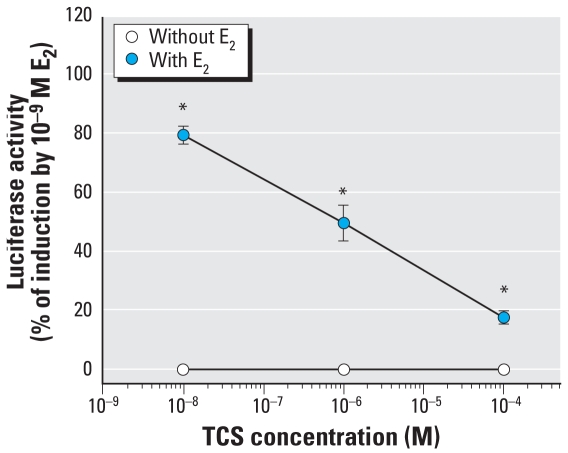
Activity of TCS in the ER-mediated bioassay. *Significantly different from the control.

The above results indicate the ability of TCC and other compounds to enhance hormone-, ER-, and AR-dependent reporter gene expression. Given the environmental and exposure relevance of TCC, we examined the concentration-dependent nature of the TCC-dependent enhancing effect in both cell bio-assay systems. We examined the effect of increased concentrations of TCC in the cell incubation medium (up to a maximum of 1 μM; we observed toxicity at higher concentrations) on ER- and AR-dependent gene expression levels using maximally inducing concentrations of E_2_ or testosterone, respectively. These analyses reveal a TCC concentration-dependent enhancement of E_2_- and testosterone-dependent reporter gene expression (Figure 5). In contrast, coincubation with TCS (VIII) resulted in a TCS concentration-dependent decrease of E_2_-dependent reporter gene expression, with 50% inhibition observed at a concentration of 1 μM TCS (Figure 6). TCS did not exhibit estrogenic activity at any concentration shown in the absence of E_2_.

### RyR-responsive bioassay

**Figure 7:**
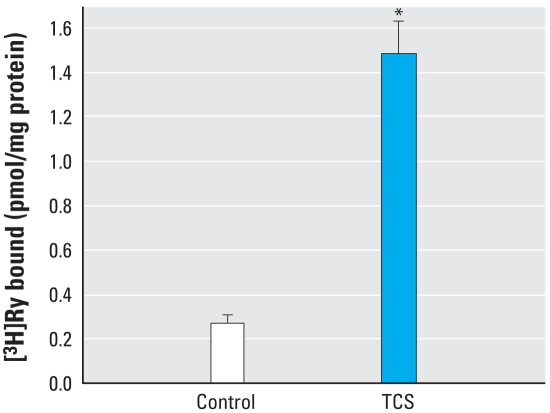
[^3^H]Ry binding with or without 1.2 μM TCS in skeletal muscle sarcoplasmic reticulum vesicles. *Significantly greater than the control at *p* < 0.05.

**Figure 8:**
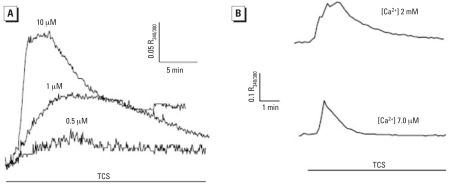
Effect of TCS on cytosolic Ca^2+^ concentration. (*A*) Cytosolic Ca^2+^ concentration in resting myotubes increased in a dose-dependent manner after TCS treatment; each trace is an average of *n* ≥ 5 cells in separate cell cultures in Ca^2+^-replete (1.8 mM) buffer. (*B*) TCS 1 μM triggered an increase in the cytosolic Ca^2+^ concentration even in nominally Ca^2+^-free (~ 7 μM) extracellular buffer.

**Figure 9:**
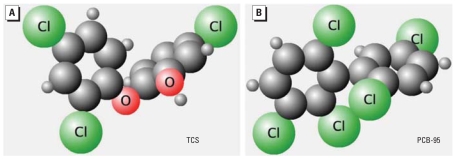
Three-dimensional projection of TCS and PCB-95 generated by ChemIDplus (National Library of Medicine 2008).

TCS significantly increased the amount of [^3^H]Ry binding to microsomes enriched in RyR1 from skeletal muscle (Figure 7). We measured net changes in intracellular Ca^2+^ concentration in myotubes with the fluorometric dye fura-2. The initial rate of Ca^2+^ increase in the cytoplasm in resting myotubes depends on the concentration of TCS (0.5–10 μM) perfused in the medium (Figure 8A). As shown in Figure 8B, TCS increased cytosolic Ca^2+^ concentrations even in the presence of a buffer containing nominally free extracellular Ca^2+^ (~ 7 μM), indicating that TCS can mobilize Ca^2+^ to the cytoplasm from endoplasmic reticulum/sarcoplasmic reticulum and/or mitochondrial intracellular stores (Figure 8B). The carbanilide compounds, including TCC, showed no significant perturbation of resting Ca^2+^ concentration when perfused at ≤ 10 μM (data not shown). TCS has a noncoplanar configuration and substitutions at the *ortho* position similar to PCB-95, one of the most active congeners toward RyR1 (Pessah et al. 2006; Wong and Pessah 1996) (Figure 9). However, TCS is significantly less hydrophobic than PCB-95 (log *p* = 4.76 vs. 6.55, respectively). Also, because of the ether linkage, TCS is significantly longer and more flexible than is PCB-95. TCS activity at RyR1 may therefore reflect its ability to assume a conformation like those of noncoplanar PCBs.

Results from molecular modeling indicate that TCS has around 67° and 100° dihedral angles formed by the two phenyl rings, similar to the configuration of PCB-95, which has around 78° and 113° dihedral angles, supporting the importance of noncoplanarity for RyR activity.

## Discussion

We have examined the biological activity of the antimicrobial TCC, its carbanilide analogs, and TCS in *in vitro* and cell-based AhR, ER, AR, and RyR bioassays. Although TCC and other carbanilide compounds exhibited no significant or weak agonist activity in the ER and AR cell bioassays, most of the compounds enhanced the ability of the steroid hormones E_2_ and testosterone to induce ER-and AR-dependent reporter gene expression in recombinant cell bioassays. In contrast, although TCS and most carbanilides, with the exception of compound VI, exhibited weak agonistic and/or antagonistic activity in the AhR-responsive cell bioassay (Table 1), none of these compounds significantly enhanced the ability of TCDD to induce reporter gene expression. These results indicate that these chemicals exhibit distinct mechanisms of action on these distinctly different ligand-dependent nuclear receptor signaling pathways.

The signal enhancement activities by the carbanilide compounds were not involved in cell proliferation. At concentrations < 1 μM, TCC, its analogs, and TCS showed no significant effect on ATP levels for cell proliferation/ cytotoxicity by the ViaLight kit in HEK-293 cells relative to the solvent control (data not shown). ViaLight cell proliferation and cytotoxicity assay was performed according to manufacturer instructions (Cambrex, Inc., East Rutherford, NJ). Similarly, < 1 μM, TCC showed no significant effect on methylthiazol tetrazolium activity in the HEK 2933Y-ARE cell proliferation assay (Chen et al. 2008). Some compounds exhibited cytotoxic effects at concentrations > 10 μM in HEK-2933Y-ARE, T47D-ARE, and BG1-ERE cell lines.

Because relatively small quantities of impurities may obscure the results for TCC, we further purified commercial TCC purchased at a purity of 99.8% by recrystallization, and we estimated higher purity (~ 100%) of TCC at 270 nm by HPLC. We evaluated the recrystallized TCC for the enhancement effect in the ER- and AR-mediated bioassays. The results were not significantly different from those in the bioassays using the commercial TCC (data not shown), so we conclude that the steroid-enhancing activity is mediated by the chemicals themselves and not by a contaminant(s).

Although the trend of the enhancing activity produced by several carbanilides in the presence of testosterone in androgen-responsive recombinant T47D breast cancer cells and HEK-2933Y cells is similar, we demonstrated the testosterone concentrations at which we observed enhanced induction to be distinctly different. The different outcomes may result from a cell-type–specific biological response for the testosterone-induced AR-dependent luciferase reporter gene expression and from differences between the endogenous AR in T47D cancer cells and the exogenous transformed AR in HEK-2933Y cells, as well as differences in the specific interlaboratory protocols.

Enhanced gene expression by carbanilide compounds in the presence of endogenous steroids in the AR- and ER-mediated reporter gene bioassays (present study) and the increased expression of AR protein by TCC and testosterone in MDA-kb2 human breast cancer cells (Chen et al. 2008) suggest that the carbanilides may sensitize the complex of receptor-associating proteins similar to cofactors or coactivators common in cells containing ER and AR (Chang and McDonnell 2005; McDonnell and Norris 2002). Such an interaction would allow the DNA-bound receptor to enhance the overall rate of transcription of the target gene as evidenced by the similar response in both ER- and AR-responsive systems. An *in vitro* AR binding assay using the rat AR ligand-binding domain and competitive fluorescence polarization measurement showed that TCC did not directly compete with testosterone for AR binding (Chen et al. 2008). However, further investigation is needed to determine whether TCC increases the activity of sex steroid hormones by binding to the receptors or to receptor coactivators.

Among the carbanilide compounds that showed gene induction enhancement, II, III (TCC), and IV are found in effluent from wastewater treatment plants; they likely result from use of personal care products or from synthesis and production of TCC or the phenylurea herbicide diuron (Sapkota et al. 2007). Carbanilide (compound I) without elemental chlorine can be obtained as a byproduct in the synthesis of the phenylurea herbicide siduron when it is prepared from phenyl isocyanate. 1,3-Dicyclohexylurea (VI) has saturated cyclohexyl rings rather than phenyl rings of compound I, and compound VI exhibited both strong agonistic and amplification activities, particularly in the ER-mediated bioassay. It is obtained in an equal molar ratio as an industrial waste by-product, along with the desired product, by dicyclohexylcarbodiimide-mediated coupling reactions for common peptide synthesis.

Hard bar soaps containing TCC, added at concentrations from about 0.6% up to 1.5%, are normally used by consumers for personal care purposes. The physicochemical properties of TCC suggest that it will penetrate the skin poorly, and this prediction is supported by limited experimental data (Halden and Paull 2005). However, soaps provide good emollients to accelerate dermal penetration of TCC. The increasing use of TCC in personal care products results in increased human exposure. Also of concern are TCC’s recalcitrance to environmental degradation and its tendency to bind tightly to organic materials such as bio-solids from sewage sludge that are reapplied to land for agriculture and other uses. Taken together, these data suggest caution in its continued use.

Possibly of broader significance is the illustration of a new mechanism of action for TCC as an EDC. The enhancement characteristics of TCC and its carbanilide analogs on endogenous/exogenous androgens and estrogens, in contrast to antagonist activity of TCS, may have an effect on possible normal physiology and/or reproduction in both males and females. Consider that synthetic and natural hormones used as growth promoters in cattle, which are eaten by women during pregnancy, have been shown to interfere with reproduction and may be involved in reduced sperm quality in their sons (Swan et al. 2007). Exposure to TCC together with the steroid hormones in women may result in similar poor sperm quality because testicular development may be altered *in utero*. An animal study has indicated that chronic oral administration of high doses of TCC to male rats resulted in testicular degeneration (Scientific Committee on Consumer Products 2005). The Hershberger assay for *in vivo* screening to evaluate the synergistic effect of testosterone by TCC demonstrated that the treatment of 0.25% (wt/wt) TCC mixed in rat chow in the presence of testosterone propionate (0.2 mg/kg by subcutaneous injection) significantly increased the weight of accessory sex organs and tissues of reproductive tracts of castrated male Sprague Dawley rats, compared with rats given testosterone treatment alone (Chen et al. 2008). Human exposure to TCC contained in commercial personal care soaps that are frequently used may enhance the activity of endogenous sex steroid hormones, suggesting that TCC as an EDC may affect male reproductive systems. In females, because the breast can be exposed to antimicrobial TCC-containing products such as soap and deodorants applied to the underarm and breast area, TCC amplification of E_2_-induced ER activity may harm patients with ER-positive breast cancer. These studies suggest the importance of evaluating the relative benefits and risks of TCC found in personal care products.

The content of TCS ranges from 0.1% to 1% in personal care products, and may reach 1% in medicated materials such as gloves/ scrubs used in hospitals where strong germicidal activity is needed. Because of its popular use, environmental residues have been detected at microgram per liter levels, suggesting extensive contamination of aquatic ecosystems and bioaccumulation in biota. Contrary to studies showing that TCS provided little or no estrogenic activity in the estrogen-dependent systems (Foran et al. 2000; Houtman et al. 2004; Ishibashi et al. 2004), we found that TCS exhibited antagonist activity in the ER-mediated bioassay (Figure 6), as it did in the AR-mediated assay (Chen et al. 2007; Tamura et al. 2006). Its phenolic structural relationship to nonsteroidal estrogens such as diethylstilbestrol and bisphenol A also raises concerns. Furthermore, TCS is structurally similar to noncoplanar *ortho*-substituted PCBs (Figure 9) and sensitizes the channel of an RyR found in various forms of muscle and other excitable animal tissue. Our results (Figures 7 and 8) are the first to identify RyRs as sensitive targets of TCS and provide a potent tool for understanding mechanisms regulating the structure and function of Ca^2+^ release complexes. These new results provide additional concern for TCS as an EDC and concern that it has potential neurotoxicity, which taken together with a finding that TCS disrupts thyroid action for normal growth and development of humans and wildlife (Veldhoen et al. 2006), suggests reevaluation of its use in consumer products.

In conclusion, we used a battery of mechanism-based screening bioassays developed by the University of California Superfund Basic Research Program for detection of toxicants and assessment of exposure to toxic substances with a selected series of carbanilides and TCS. As summarized in Table 2, we found that a group of carbanilides, including the antimicrobial TCC, enhances effects of steroid hormones in ER- and AR-dependent gene expression bioassays, although the compounds have minor endocrine activity on their own, suggesting a new class of EDC. In general, the carbanilide compounds except for dicyclohexylurea (VI), which acted as an agonist, provided antagonistic activity in the AhR-responsive cell bioassay. Similar to noncoplanar *ortho*-substituted PCBs, the noncoplanar antimicrobial compound TCS exhibited weak AhR activity but was a potent antagonist in both ER-mediated and AR-mediated bioassays and a potent channel sensitizer in an RyR-mediated bioassay and dysregulator of cell Ca^2+^ homeostasis. These observations have potentially significant implications regarding human and animal health because exposure may be directly through dermal contact or indirectly through the food chain. These screening studies revealed that further investigations into the biological and toxicologic effects of TCC, its carbanilide analogs, and TCS are urgently needed.

## Correction

In Figure 2 of the original manuscript published online, the concentration of TCC was incorrect; it has been corrected here.

## Figures and Tables

**Table 1:** Induction or inhibition of AhR-dependent luciferase reporter gene expression in H4L1.1c4 cells.

	Luciferase activity (percent of TCDD)	
Compound	1 μM of compound	10 μM of compound
Induction of luciferase		
TCDD	100 ± 8	100 ± 8
I	2.0 ± 0.1	1.8 ± 0.2
II	0.1 ± 1.7^a^	0.6 ± 0.1^a^
III (TCC)	0.8 ± 0.4^a^	1.2 ± 0.3^a^
IV	0.1 ± 0.2^a^	0.1 ± 0.5^a^
V	0.5 ± 0.7	1.3 ± 0.4
VI	50.9 ± 1.5*	13.6 ± 1.9
VII	8.5 ± 0.6	3.6 ± 0.2*
VIII (TCS)	6.0 ± 1.5	40.6 ± 6.1*
Inhibition of TCDD induction of luciferase		
DMSO	100 ± 8	100 ± 8
I	85.9 ± 4.4*	80.3 ± 0.6^a^*
II	61.4 ± 0.7^a^*	81.3 ± 4.4^a^*
III (TCC)	64.6 ± 3.2^a^*	50.7 ± 2.8^a^*
IV	75.1 ± 2.5^a^*	33.4 ± 4.1^a^*
V	98.2 ± 3.2	79.3 ± 4.6*
VI	103 ± 2	112 ± 3
VII	97.1 ± 2.0	32.5 ± 1.6*
VIII (TCS)	98.3 ± 5.5	70.4 ± 2.1*

Values are expressed as a percentage of that induced by 1 nM TCDD and represent the mean ± SD of triplicate determinations of luciferase activity.

a Data from Zhao et al. (2006).

* Significantly different from the DMSO-treated controls or TCDD-treated samples (*p* < 0.05).

**Table 2:** The biological activity of TCC, its analogs, and TCS in the receptor bioassay screens.

	AhR (H4L1.1c4-DRE cells)		ER (BG1-ERE cells)		AR (T47D-ARE or HEK2933Y-ARE cells)		
Compound	Compound alone	With 1 nM TCDD	Compound alone	With 1 nM E2	Compound alone	With 0.1 nM or 10 μM T	RyR ([^3^H]Ry binding)
I	–	+ (ant)	+ (ag)	+ (amp)	+ (ag^a^)	+ (amp^a^,^b^)	–
II	–	+ (ant)	+ (ag)	+ (amp)	+ (ag^a^)	+ (amp^a^,^b^)	–
III (TCC)	–	+ (ant)	+ (ag)	+ (amp)	–	+ (amp^a^,^b^)	–
IV	–	+ (ant)	+ (ag)	–	–	+ (amp^a^,^b^)	–
V	–	+ (ant)	+ (ag)	+ (amp)	− (ag^a^)	+ (amp^b^)	–
VI	+ (ag)	–	+ (ag)	+ (amp)	–	+ (amp^a^)	–
VII	+ (ag)	+ (ant)	+ (ag)	+ (amp)	–	–	–
VIII (TCS)	+ (ag)	+ (ant)	–	+ (ant)	–	+ (ant^b^)	+ (sensitive)

Abbreviations: –, no effect; +, positive effect; ag, agonistic; amp, amplification; ant, antagonistic.

a Data from T47D-ARE bioassays.

b Data from HEK 2933Y-ARE bioassay.
